# Agent-based simulations for protecting nursing homes with prevention and vaccination strategies

**DOI:** 10.1098/rsif.2021.0608

**Published:** 2021-12-22

**Authors:** Jana Lasser, Johannes Zuber, Johannes Sorger, Elma Dervic, Katharina Ledebur, Simon David Lindner, Elisabeth Klager, Maria Kletečka-Pulker, Harald Willschke, Katrin Stangl, Sarah Stadtmann, Christian Haslinger, Peter Klimek, Thomas Wochele-Thoma

**Affiliations:** ^1^ Institute for Interactive Systems and Data Science, Graz University of Technology, Graz, Steierermark, Austria; ^2^ Institute for Interactive Systems and Data Science, Graz University of Technology, Graz, Austria; ^3^ Complexity Science Hub Vienna, Wien, Austria; ^4^ Medical University Vienna, Section for Science of Complex Systems, Wien, Austria; ^5^ Research Institute of Molecular Pathology, Wien, Austria; ^6^ Ludwig Boltzmann Institute for Digital Health and Patient Safety, Vienna, Austria; ^7^ University Vienna, Institut für Ethik und Recht in der Medizin, Wien, Austria; ^8^ Caritas Erzdiözese Wien, Wien, Austria; ^9^ Hygienefachkraft-unlimited, Wien, Austria

**Keywords:** agent-based simulations, long-term care facilities, nursing homes, mitigation testing, infection dynamics, non-pharmaceutical interventions

## Abstract

Due to its high lethality among older people, the safety of nursing homes has been of central importance during the COVID-19 pandemic. With test procedures and vaccines becoming available at scale, nursing homes might relax prohibitory measures while controlling the spread of infections. By control we mean that each index case infects less than one other person on average. Here, we develop an agent-based epidemiological model for the spread of SARS-CoV-2 calibrated to Austrian nursing homes to identify optimal prevention strategies. We find that the effectiveness of mitigation testing depends critically on test turnover time (time until test result), the detection threshold of tests and mitigation testing frequencies. Under realistic conditions and in absence of vaccinations, we find that mitigation testing of employees only might be sufficient to control outbreaks if tests have low turnover times and detection thresholds. If vaccines that are 60% effective against high viral load and transmission are available, control is achieved if 80% or more of the residents are vaccinated, even without mitigation testing and if residents are allowed to have visitors. Since these results strongly depend on vaccine efficacy against infection, retention of testing infrastructures, regular testing and sequencing of virus genomes is advised to enable early identification of new variants of concern.

## 1. Introduction

Nursing homes and other long-term care facilities are the ground zero of the COVID-19 pandemic [1]. Around the globe, a disproportionate number of confirmed deaths has been attributed to nursing home residents. For instance, as of July 2020, nursing homes accounted for 37% of the 719 confirmed COVID-19 deaths in Austria [2]. With 923 confirmed cases in nursing homes during this period of time, this results in a case fatality rate of 28%, in line with reported high case fatality rates in the age group above 80 years old [3].

Owing to this extreme severity, in most countries stringent non-pharmaceutical interventions have been suggested for nursing homes, such as bans on visitors, individual movement restrictions and other quarantine policies [4,5]. COVID-19, therefore, severely affects the quality of life of all nursing home residents, not just the infected ones [6].

The widespread availability of rapid testing procedures, e.g. antigen tests [7] or tests based on the RT-LAMP procedure [8] enable ‘testing for mitigation’ strategies. The aim of mitigation testing is to use testing within a specific setting to quickly identify and isolate infectious individuals but, in contrast to diagnostic testing, not necessarily infected individuals which do not have a high enough viral load to infect others. As vaccines become available, nursing homes naturally have become priority targets for the vaccination of as many employees and residents as possible [9,10].

At the moment, non-pharmaceutical measures, testing strategies and vaccines exist side-by-side. In the intermediate future, vaccines will most likely replace non-pharmaceutical measures and reduce the need for testing. Yet, at this point in time, it is unclear how to design optimal mitigation strategies involving testing and vaccination. Moreover, a transition from purely non-pharmaceutical interventions over a mix of vaccines and interventions towards widespread vaccine proliferation is expected to occur for potential future immune escape variants of SARS-CoV-2 and any other pandemic threat yet to emerge.

The design of optimal mitigation testing strategies for a specific facility like nursing homes is challenging due to the following and sometimes interrelated factors that determine the effectiveness of a given strategy [11]: next to the epidemiological contagion dynamics, the optimal testing strategy also depends on the structure of the co-location networks of the employees and residents, personal protective and physical distancing measures already in place, as well as the characteristics of the test, namely (i) the turnover time (time span between test and availability of test result) and (ii) the detection threshold (viral load necessary for a positive result). For instance, RT-PCR tests, the current gold standard, have a turnover time of 1 or 2 days with a detection threshold much lower than the threshold above which an individual becomes infectious. Antigen tests have a turnover of less than an hour but a substantially higher detection threshold. Finally, RT-LAMP tests combine a same-day turnover with a low detection threshold. For all these tests, sensitivity and specificity are close to 100% above the corresponding detection thresholds [12–14].

First evidence shows that vaccinations can be highly effective in preventing severe courses of the disease [15], infection [16–20] and in reducing onward transmission [21,22]. Given the impact of measures such as a reduction in visits and physical distancing on the mental and emotional wellbeing of residents [23], ethical questions concerning the necessity of non-pharmaceutical interventions alongside vaccinations arise. In the context of substantial vaccine hesitancy among employees [24,25], an assessment of the necessary level of non-pharmaceutical intervention measures given different levels of vaccination prevalence and effectiveness is crucial.

**Figure 1.**
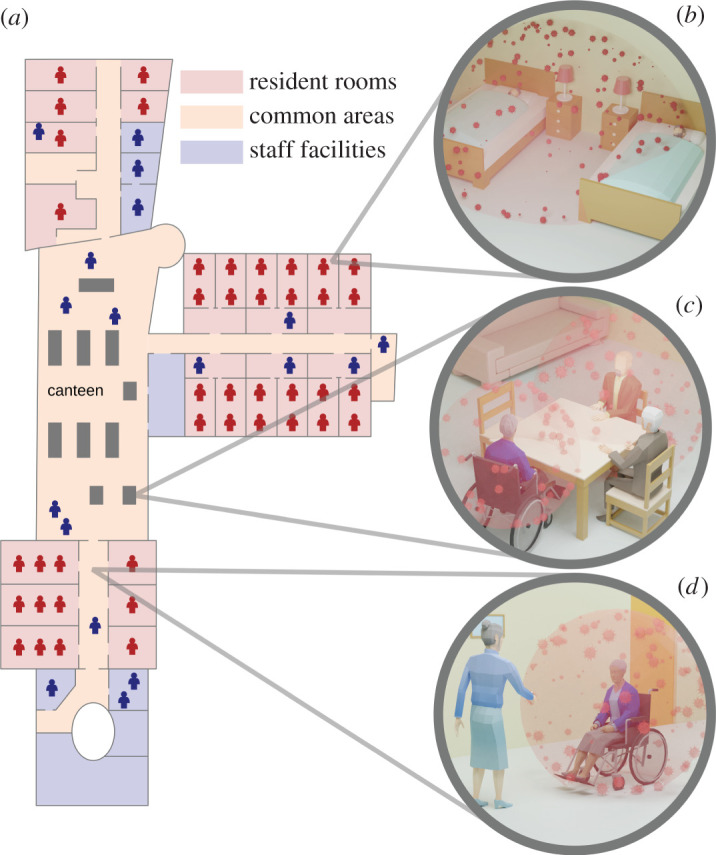
Living conditions in a ward of an Austrian nursing home. (*a*) Simplified floor plan of the ward with resident rooms (red), common areas (orange) and staff facilities (blue). The ward houses up to 42 residents (red figures), is staffed by 18 employees (blue figures) and corresponds to the homes described in case studies 2 and 3 (see electronic supplementary material, note S5). Contact networks for the simulations were extracted from such floor plans and information about shared tables in the canteen. (*b*) Rooms: up to two residents share a room and up to two rooms share a bathroom. (*c*) Shared table: up to six residents share a table during joint meals. (*d*) Shared common areas: residents living in the same ward of the home can move freely within the hallways, canteen and other common areas and regularly meet other residents. Spread of the virus by means of aerosols [36] is indicated as red clouds.

Here, we aim to design optimal mitigation testing and vaccination strategies for nursing homes for different testing technologies and levels of vaccination prevalence by means of network-based epidemiological modelling [26]. In particular, we use an SEIRX model that is calibrated with individual-level data from Austrian nursing homes. Individuals start susceptible (S). After exposure (E), they turn infectious (I) and recover after some time (R). Depending on the test technology, individuals can be tested positive either before or after becoming infectious. Vaccinations lower both the chance of infection and transmission. If infected individuals are identified by a test, they are isolated (X). Individual-based epidemiological models with a similar structure have already been used to investigate the spread of SARS-CoV-2 in nursing homes under different prevention measures such as routine testing [27–30], and the combination of testing with vaccination strategies [31–34]. Overall, these previous studies have shown that routine testing needs to be frequent enough to mitigate outbreaks and that this frequency can be reduced in proportion to vaccination rates in employees and residents. Contagions occur on a network of social contacts [35] that is modelled after the actual living conditions in a nursing home (figure 1). We consider residents and employees with different types of social interaction such as shared rooms or living and working in the same ward of a nursing home. We use empirical data from four outbreaks to calibrate the transmission risk associated with different types of interactions (see electronic supplementary material, note S3). Simulations were calibrated using data of outbreaks involving the wild-type SARS-CoV-2 strain that was dominant in Austria in spring 2020. Since then, the B.1.1.7 (alpha) variant has become dominant, which is reported to have a 50% increased transmissibility [37–41]. We model this variant in all results presented in this work by increasing the transmissibility that was calibrated using wild-type outbreak data by 50%. For results of outbreaks with the wild-type strain, see electronic supplementary material, note S6.

Mitigation testing strategies are parametrized by test technology and testing frequency. We consider RT-PCR and antigen tests with their specific turnover times and detection thresholds. Additional results for RT-LAMP tests are shown in electronic supplementary material, note S8. Furthermore, the strategies are determined by the frequencies by which all residents and employees are tested, respectively. Vaccination prevalence is parameterized by the ratio of vaccinated employees and residents. We assume that vaccinations are 60% effective in preventing infection and 30% effective in preventing transmission. These are conservative estimates of the vaccine efficacies reported three or more weeks after the first dose of the BNT162b2 (Biontech-Pfizer) and ChAdOx1 nCOV-19 (AstraZeneca) vaccines, which are currently most prevalent in Austrian nursing homes [16–19,21,22].

Here, we investigate the effectiveness of different testing and vaccination strategies in preventing infections in nursing homes given the presence of an infected resident or employee. For a given set of prevention measures, we compute the expected distributions of outbreak sizes amongst the residents, i.e. the average number of infected residents after introducing a single index case into the home and simulating the ensuing outbreak. In particular, we assume that index cases are introduced either through an employee or a resident from outside the home. The former reflects a situation where visitors are not allowed and residents' contacts with individuals from outside the nursing home are limited. The latter reflects a situation where residents can introduce an infection into the home, for example through contact with visitors. In the absence of vaccinations, the optimal testing strategy is then identified as the test technology and test frequency for residents and personnel that minimizes the outbreak size for a given number of tests being performed. If an index case causes fewer than one infection among residents, we label the situation as ‘controlled’. For scenarios in which a number of employees and residents are vaccinated, we investigate how much testing is necessary to achieve the same level of security as the optimal testing strategy.

## 2. Methods

We simulate the infection dynamics using an agent-based model. The model includes two types of agents (residents and employees) that live and work in nursing homes, respectively. Infections are introduced from outside the home either through an employee or a resident (see electronic supplementary material, note S1 ‘Index cases').

Residents have individual networks of social contacts. The co-location network defines interactions between residents in one of two ways, in decreasing order of infection transmission risk: two residents might have social contacts due to a shared room, a shared meal table, or a shared ward. While occasional contacts between different wards of the same facility are possible, we assume wards to function independently from each other. The co-location network used in our simulation is a model of social contacts in a nursing home ward, based on data about occupancy and staffing during a number of observed outbreaks in homes, and information from practitioners (see electronic supplementary material, note S1 ‘Co-location networks'). One ward includes 35 residents and 18 employees, resembling a typical ward in an Austrian nursing home (see electronic supplementary material, note S5 for details).

At the first day of a simulation, a random resident or employee is chosen to become the index case and the agent's state is set to ‘exposed’. At every step (day) of the simulation, agents interact according to their interaction rules and infectious agents can transmit the virus to susceptible individuals. Depending on an agent's individual exposure duration, incubation duration, infection duration and probability to develop symptoms (see electronic supplementary material, note S1 ‘Agents’), each agent is in one of 12 states: susceptible (*S*), exposed (*E*), infectious presymptomatic (*I*), infectious asymptomatic (*I*_1_), infectious symptomatic (*I*_2_) or recovered (*R*) (depicted in electronic supplementary material, figure 6B). Each of these states also exists in an isolated/quarantined (*X*) version). States *S*, *E*, *I* and *R* also translate to viral load, as depicted in electronic supplementary material, figure 6A, which is important for the ability of different test technologies to detect an infection (see electronic supplementary material, note S1 ‘test technologies’). Once an agent has become infected, the agent stays exposed for an average of 5 days, matching the latent time reported for SARS-CoV-2 [42,43]. After 5 days on average, agents become infectious and stay infectious for on average 11 days [44,45].

The risk of transmission to a contact person is particularly high during the first days of the infectious phase and then decreases as the infection progresses [44,46]. Not all agents develop symptoms and the probability to develop symptoms depends on age [47]. We assume that the age of employees is uniformly distributed between 20 and 59 years. Therefore, employees have an average probability to develop symptoms of 26.46% [47]. For residents, we assume a probability to develop symptoms of 64.52%, corresponding to the value reported by Poletti *et al*. for people aged 80 and above. We do not consider age to be a relevant factor for susceptibility, since evidence for this effect is still inconclusive [48]. If an agent develops a symptomatic course of the disease, symptoms start to appear shortly after becoming infectious [46]. Transmissibility of asymptomatic agents is reduced by 40% [49]. Vaccinations reduce the susceptibility by 60% [16–19], and the transmissibility by 30% [21,22]. These are conservative estimates of the values reported in the literature for the vaccines BNT162b2 and ChAdOx1 nCOV-19 that are predominantly used in Austrian nursing homes.

In our model, each time-step (day) of the simulation is associated with an independent Bernoulli trial for disease transmission between susceptible and infectious agents given a contact [50,51]:

$$P=1-[1-\beta (1-q_{1}(c))(1-q_{2}(t))(1-q_{3})(1-q_{4})(1-q_{5})],$$

where *β* is the transmission probability per person per day, calibrated to reflect the secondary attack rate for household contacts between adults of 28.3% [48] (see electronic supplementary material, note S3). The modifier *γ* *=* 1.5 reflects the 50% increase in transmissibility reported for the alpha virus variant [37–41]. The *q_i_* are reductions of transmission risk due to the various factors described above: *q*_1_(*c*) modifies transmission risk depending on contact type *c, q*_2_(*t*) reflects the reduction of transmission risk due to lower viral loads as the infection progresses, *q*_3_ reflects reduced transmissibility due to asymptomatic presentation, and *q_4_* and *q_5_* represent reduced susceptibility and transmissibility due to vaccinations (see also electronic supplementary material, note S1 ‘Transmission probability’). We consider the use of masks by employees in a separate model described in electronic supplementary material, note S1 ‘Other intervention measures'.

We calibrate *β* and *q_1_* by means of an iterated grid search such that the transmission risk for close contacts reflects the household secondary attack rate, and such that outbreak sizes produced by our model correspond to observed outbreak sizes in nursing homes. All other *q_i_* are chosen to correspond to values reported in the literature. See electronic supplementary material, note S1 for details of model design, implementation and assumptions, electronic supplementary material, note S2 for an overview of all model parameters and their sources and electronic supplementary material, note S3 for details about the calibration.

Exposed or infectious agents can be testable and return a positive result when tested, depending on the period of time they have already been infected and the test being used (electronic supplementary material, figure 6A). For sake of simplicity, we model the time-dependent sensitivity of all simulated tests by assuming that there is a limited time window within which they detect an infection (or its absence) with certainty and by varying the onset and duration of this time window between different types of test (see electronic supplementary material, note S1 ‘Test technologies’ for details). In the baseline scenario, only diagnostic testing takes place: symptomatic cases are immediately isolated and tested using a PCR test with a 2-day turnover time. Once a positive test result is returned, all close and intermediate contacts of the positive agent are immediately quarantined but not tested. We summarize this strategy as ‘test–trace–isolate’ (TTI) (details in electronic supplementary material, note S1 ‘Intervention measures'). In addition, nursing homes can implement a ‘mitigation testing strategy’ where they test all their employees and/or residents with a given testing frequency, regardless of symptomatic cases, i.e. a symptomatic case still triggers the TTI process but the mitigation testing is not influenced by it. Simulations start at a random day with respect to the time interval until the first mitigation test is performed, i.e. a maximum of 6 days, if mitigation testing is performed once every 7 days. Both TTI and mitigation testing are performed irregardless of the vaccination state of an agent.

To model vaccinations, for a given vaccination ratio in an agent group, a number of agents from that group corresponding to the ratio are picked at random at the start of the simulation and assigned a vaccination state. Being vaccinated reduces both the probability for a successful transmission to a vaccinated and susceptible agent (*q*_4_), and from a vaccinated but infected agent to another susceptible agent (*q*_5_). We also model protective equipment for employees; see electronic supplementary material, note S1 ‘Other intervention measures') for details.

## 3. Results

We simulate epidemic spread in a nursing home in a range of different scenarios: (i) introduction of index cases through either employees or residents, (ii) different testing technologies used for mitigation testing, (iii) different intervals for the mitigation tests, (iv) different prevalence of vaccinations and (v) combinations of different testing strategies and vaccinations.

Next to the average outbreak size, we report the average number of transmissions from the index case, the reproduction number *R*_eff_, [52] and the total number of tests per day per person needed to implement the testing strategy (test rate).

### 3.1. Effectiveness of test–trace–isolate

In the absence of non-pharmaceutical interventions or containment measures and infection with the alpha variant, we find *R*_eff_ = 2.25 [0; 7] (mean [2.5% quantile; 97.5% quantile]) if an employee is the index case and *R*_eff_ = 2.97 [0; 8] if a resident is the index case. Mean outbreak sizes are 25.2 ± 13.9 if an employee is the index case and 26.8 ± 11.7 if a resident is the index case. In a scenario in which only TTI is implemented, our model yields reproduction numbers of *R*_eff_ = 1.90 [0; 6] if an employee is the index case, and *R*_eff_ = 1.82 [0; 6] if a resident is the index case and mean outbreak sizes of 13.8 ± 11.9 and 12.6 ± 11.7 for employee and resident index cases, respectively.

### 3.2. Effectiveness of different testing strategies

Mean outbreak sizes range between 0.1 ± 0.5 and 14.0 ± 11.9. Higher testing frequencies and lower test turnover times always reduce the size of outbreaks. Intuitively, prioritizing the agent group that is more likely to introduce index cases in the mitigation testing strategy considerably reduces the size of outbreaks. The lowest outbreak sizes of 0.1 ± 0.5 are achieved if index cases are predominantly introduced by employees and residents and employees are tested three times a week with PCR tests with same-day turnover. The highest outbreak sizes of 14.0 ± 11.9 are recorded if no regular mitigation testing happens and an employee is the index case. Nevertheless, it is noteworthy that a TTI strategy using PCR tests with a 2-day turnover is sufficient to contain outbreak sizes (i.e. the infection is stopped in the majority of cases before all 35 residents are infected) in these scenarios.

**Figure 2.**
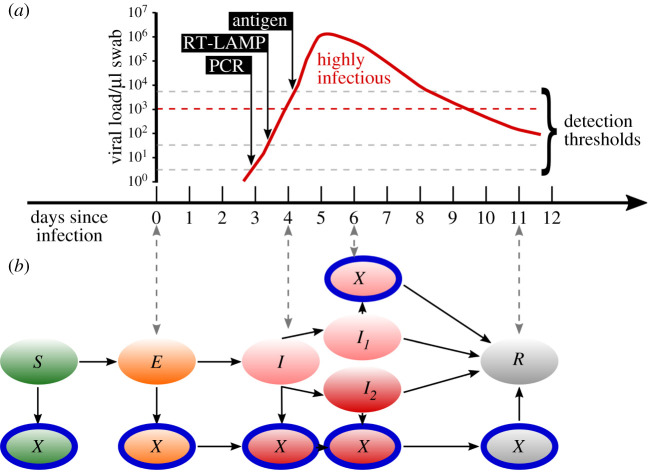
Testability and agent states of the agent-based epidemiological model. (*a*) Illustration of viral load over time and detection thresholds of PCR, RT-LAMP and antigen tests reproduced after Kellner *et al*., [13]; Larremore *et al*., [53]; Wölfel *et al*., [45]: in our model, PCR tests can detect an infection 1 day before an agent becomes infectious, RT-LAMP tests on the day an agent becomes infectious and antigen tests 1 day after an agent becomes infectious. Individuals with greater than 10^3^ virus copies per microlitre swab are considered infectious [45]. (*b*) Agents in the epidemiological model can be in the states (circles) susceptible (*S*), exposed (*E*), infectious presymptomatic (*I*), infectious asymptomatic (*I1*_1_), infectious symptomatic (*I*_2_) and recovered (*R*). Possible state transitions are shown by arrows. Each of these states also exists in an isolated/quarantined version (*X*), preventing an agent from interacting with other agents. Transitions between states follow the individual agent's exposure durations, incubation times and infection durations.

We report the main results for a realistic scenario where index cases are introduced by employees (because residents are not allowed to have visitors), employees are tested twice per week while residents are not tested at all, and PCR tests achieve a turnover time of 1 day. Mean outbreak sizes (infected residents) and their standard deviation for this scenario for both resident and employee index cases are shown in figure 2. The results for different testing strategies are reported in electronic supplementary material, figures A7 and A8 in the appendix PCR tests clearly outperform antigen tests, with outbreak sizes of 1.5 ± 4.7 for an employee index case and 6.1 ± 7.6 for a resident index case (PCR), and 3.1 ± 6.3 and 7.1 ± 8.3 for employee and resident index cases, respectively (antigen). If the logistics around PCR tests are optimized such that a same-day turnover can be achieved, PCR perform even better with outbreak sizes of 0.9 ± 3.7 (employee) and 5.6 ± 7.3 (resident). If employees are tested only once a week, outbreak sizes increase to 3.0 ± 6.6 and 7.0 ± 8.4 (PCR, same-day turnover) and 5.8 ± 9.0 and 8.9 ± 9.5 (antigen) for employee and resident index cases, respectively. *R*_eff_ for each scenario is shown in electronic supplementary material, figure A9. For mean and median outbreak sizes alongside the 10th and 90th percentile of the outbreak size distribution, R_eff_, and test rates for each of the three test technologies with same-day turnover, as well as 1-day and 2-day turnover for PCR tests, testing of employees never, once, two times or three times a week and testing of residents never or once a week, see electronic supplementary material, note S4.^1^ If, in addition to the testing, employees wear protective equipment, this offsets the increased transmissibility of the alpha variant and reduces outbreak sizes to sizes comparable to the ones observed for the wild-type (see electronic supplementary material, notes S6 and S7).

The base rate of tests needed for TTI in all scenarios is approximately 0.003 ± 0.002 tests per day per person. Implementation of regular mitigation testing of only employees two times a week increases this rate to 0.09 ± 0.013, independent of test technology and index case. Implementation of regular testing twice per week for only residents increases the rate to 0.18 ± 0.02. Implementation of tests three times a week increases the rate to 0.27 ± 0.03. The test rate for each scenario is visualized in electronic supplementary material, figure A10.

**Figure 3.**
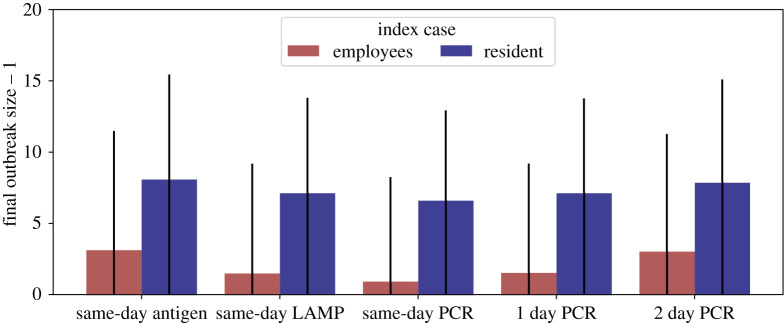
Outbreak sizes for different test technologies and result turnover times. Outbreak sizes are calculated as the final number of infected residents. If the index case was a resident, one is subtracted from the final outbreak number. Outbreak sizes are shown for a realistic mitigation testing scenario in which employees are tested twice per week but residents are not tested. Mean outbreak sizes and standard deviations (black bars) are shown for employee (red) and resident (blue) index cases for five different test technology and test turnover combinations. Outbreak sizes for each combination are averages over 5000 randomly initialized simulation runs each. In addition to mitigation testing, the model also implements TTI.

In figure 3, we investigated different test turnover times between same-day and 2 days for PCR tests in the same model setting as described above. In the same scenario as described above (employee testing twice per week, employee as index case), and in case of PCR tests with a turnover rate of 2 days, mean outbreak sizes increase significantly to 3.0 ± 6.5 (employee index case) and 6.9 ± 8.2 (resident index case) – very similar to the performance of the less accurate antigen tests. Only if employees are tested three times a week and residents are tested at least twice a week, outbreak sizes drop to 0.5 ± 1.4 (employee index case) and 0.9 ± 1.9 (resident index case). PCR tests with same-day turnover are obviously the best option and reduce outbreak sizes to 0.8 ± 2.0 (employee index case) and 0.9 ± 2.0 (resident index case), even if employees and residents are tested only once a week. *R*_eff_ and test rates for different PCR test turnover times are visualized in electronic supplementary material, figures A11 and A12.

### 3.3. Effectiveness of vaccinations

**Figure 4.**
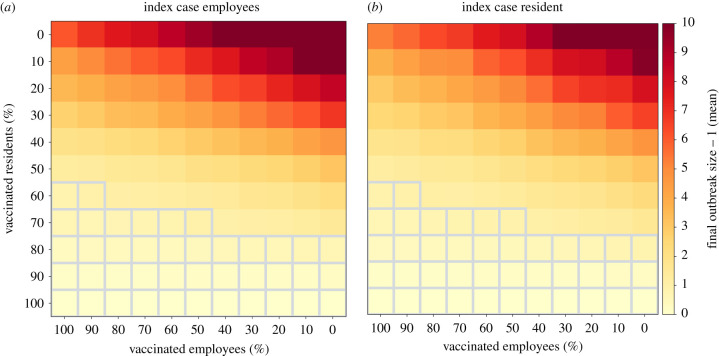
Outbreak sizes for different ratios of vaccinated employees and residents. Outbreak sizes are indicated from low (yellow) to high (red) for (*a*) employee index cases and (*b*) resident index cases. Vaccination ratios for which the mean number of resident follow-up cases is less than 1 are indicated with grey borders. Outbreak sizes for each combination of (employee, resident) vaccination ratio are averages over 5000 randomly initialized simulation runs each. In addition to vaccinations, the model also implements TTI.

To assess the impact of vaccination prevalence on outbreak sizes, we simulate scenarios with different vaccination rates for employees and residents. Additionally, nursing homes implement only TTI, i.e. testing and isolating symptomatic agents and their contacts, but no mitigation tests. For a scenario in which vaccinations are scarce and employees are prioritized for vaccinations (50% of employees are vaccinated), outbreak sizes range from 9.4 ± 9.8 (employee index case) to 7.0 ± 9.3 (resident index case). If residents are prioritized instead, outbreak sizes are reduced to 3.1 ± 4.1 and 3.1 ± 4.0, respectively. In a situation in which the vaccine is broadly available but employees are hesitant to get vaccinated (50% of employees and 90% of residents vaccinated), outbreak sizes are reduced to 0.2 ± 0.5, independent of the index case. Interestingly, further increasing the ratio of vaccinated employees only slightly reduces the number of follow-up cases among residents, compared to the scenario in which employees show vaccine hesitancy: if 90% of residents and employees are vaccinated, outbreak sizes are 0.1 ± 0.5, independent of the index case. In figure 4, we show outbreak sizes for a wide range of vaccination rates. In general, if 80% or more of the resident population is vaccinated, outbreak sizes stay less than 1, independent of the number of vaccinated employees and outbreaks are controlled. In electronic supplementary material, note S6 we report similar simulation results for the strain thatwas dominant in Austria during data collection. For this less infectious strain, it is sufficient if 60% or more of the resident population is vaccinated to keep outbreak sizes below one. In electronic supplementary material, note S7, we report the same results for a scenario in which B1.1.7 is introduced to the nursing home and employees are required to wear protective gear. We find that in this scenario a vaccination rate of 70% amongst employees is sufficient to control the spread of the more transmissible variant. If a high number of residents is vaccinated, vaccinating an increasing number of employees is still beneficial, since it further lowers the number of infected residents towards zero (see electronic supplementary material, figure A13).

To assess the impact of vaccination rates on the merits of different testing strategies, we simulate scenarios in which parts of the nursing home population are vaccinated while different testing strategies are employed at the same time. We specifically analyse three scenarios: (i) employees are subject to frequent mitigation testing (two times per week) with cheap, fast but insensitive antigen tests on top of TTI, (ii) employees are subject to frequent mitigation testing with expensive, fast and very sensitive PCR tests on top of TTI and (iii) there are no mitigation tests and homes rely solely on vaccines and TTI to prevent the spread of infections.

**Figure 5.**
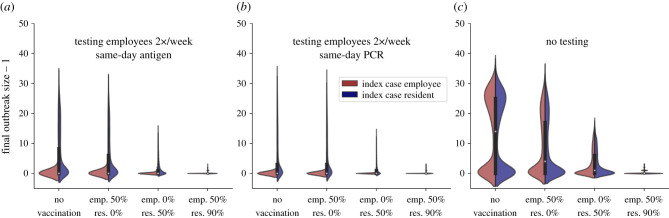
Distributions of the number of infected residents for different vaccination scenarios and testing strategies. The left (red) part of the violins indicates employee index cases, the right (blue) part of the violins indicates resident index cases. (*a*) The testing strategy consists of TTI and preventive testing of employees two times a week with antigen tests with a same-day turnover. (*b*) The testing strategy consists of TTI and preventive testing of employees two times a week with PCR tests with same-day turnover. (*c*) The testing strategy consists of TTI only. For every testing strategy, four vaccination scenarios (no vaccination, 50% of employees, 50% of residents, 50% of employees and 90% of residents) are shown. The plot shows distributions of outbreak sizes from 5000 randomly initialized simulation runs per testing strategy and vaccination scenario.

If 50% of the resident population is vaccinated, same-day turnover antigen and PCR tests perform similarly well, with outbreak sizes of 0.5 ± 1.6 (antigen, employee index case) and 1.6 ± 2.5 (antigen, resident), and 0.9 ± 3.7 (PCR, employee) and 1.3 ± 2.1 (PCR, resident). If no residents are vaccinated, only mitigation testing with highly sensitive tests with a fast result turnover can keep outbreak sizes in check: if employees are not vaccinated as well, we observe outbreak sizes of 0.9 ± 3.7 (employee) and 5.6 ± 7.3 (resident) if employees are tested twice a week with same-day turnover PCR tests. Using the same test setup, if 50% of employees are vaccinated, outbreak sizes are only mildly reduced, with 0.8 ± 3.3 and 5.2 ± 6.9 for employee and resident index cases, respectively. If 50% of employees and 90% of residents are vaccinated, additional testing only slightly reduces outbreak sizes further, since outbreak sizes are already very low: no testing yields outbreak sizes of 0.2 ± 0.5 (independent of the index case), testing employees twice a week with antigen tests yields outbreak sizes of 0.0 ± 0.3 and 0.1 ± 0.4 and testing employees twice a week with PCR tests yields outbreak sizes of 0.0 ± 0.2 and 0.1 ± 0.5 for employee and resident index cases, respectively—ensuring almost a complete stop of any transmissions. We show outbreak size distributions for all three selected testing strategies and all four vaccination scenarios in figure 5. In electronic supplementary material, figure A14 results for all previously discussed testing frequencies and vaccination scenarios are shown.

## 4. Discussion

In this study, we aimed to design optimal prevention measures for nursing homes by means of an agent-based epidemiological model. The model reflects individual-level co-location networks modeled after the living conditions in a nursing home. Epidemic dynamics have been calibrated to recorded outbreak events. By considering three different testing technologies, we identified testing frequencies for residents and employees that result in the minimal average outbreak size at a given maximal capacity to perform tests. By considering three realistic vaccination scenarios together with various testing strategies, we identify the optimal prevention strategy depending on vaccine availability.

In brief, our simulations confirm that diagnostic testing of residents and employees combined with quarantine of close contacts of positive cases (TTI) according to current recommendations for nursing homes [54] limits outbreak sizes in nursing homes to approximately 13 follow-up cases per index case in situations where the more transmissible alpha variant is dominant. Compared to the baseline TTI scenario, more frequent testing, faster turnover of the test results, and a lower detection threshold for the tests are always beneficial to reduce the average outbreak size. However, the extent to which these individual factors contribute to an outbreak size reduction is non-trivial.

For scenarios in which contacts between residents and visitors or other external people are drastically reduced and we can assume that infections are introduced into the home solely through employees, we find that the marginal effectiveness (outbreak size reduction per performed test) of personnel testing strongly outperforms the marginal effectiveness of resident testing. This means that testing only the personnel two or three times per week can have an equal or even higher protective effect than testing all residents once per week. However, in cases where infections can be introduced through residents, i.e. residents frequently have visitors, these visits take place without other precautionary measures and the visitors have a high risk to be infected themselves, also testing of residents becomes increasingly important.

All of our results are strongly sensitive to the turnover time between the test being performed and the arrival of the test result. Reducing this timespan from 2 days to a same-day turnover might reduce the average outbreak size from around 3.0 ± 6.5 follow-up cases per index case to 0.9 ± 3.6 cases, in a scenario where personnel is regularly tested twice a week with PCR tests and index cases are introduced by employees. Such a strategy becomes increasingly feasible as point-of-care PCR tests become available [55]. Depending on the scenario, antigen tests yield between 0.1 ± 0.3 and 0.4 ± 0.8 false-negative tests (per outbreak), which has dramatic consequences, since the false-negative person is not isolated and is able to freely spread the infection.

In our model, we do not consider how convenient it is to be tested with a given method. Many PCR and antigen tests require a throat swab that can become quite a nuisance, particularly if employees have to undergo this procedure twice a week. In addition, staff in Austrian nursing homes report that older people, often living with dementia, do not respond well to the often-painful testing. PCR tests can be performed by gargling a tasteless liquid which might be beneficial for long-term compliance with the testing regimen.

To simulate the effect that varying amounts of vaccinated residents and employees have on the infection dynamics in homes, we assumed vaccine efficacies of 60% to prevent infection and of 30% to prevent transmissions. Given these rather conservative estimates of vaccine efficacy, if 80% of residents are vaccinated an index case in a nursing home leads to less than one follow-up case, even if no employees are vaccinated and homes only perform TTI. In a scenario where 90% of residents are already vaccinated, further increasing the ratio of vaccinated employees is still beneficial, as it further reduces the average number of infected residents: if 90% of employees are vaccinated, the number of infected residents is reduced to 0.13 ± 0.39, as compared to 0.17 ± 0.46 if only 50% of employees are vaccinated (employee index case).

If high numbers of residents are vaccinated, mitigation testing only slightly reduces outbreak sizes further and there is no significant difference between the introduction of the index case by an employee or resident. This means that if the resident population is vaccinated to a high degree (more than 80%), expensive and logistically complex mitigation testing schemes at scale can be discontinued without risking large outbreaks, and there is no justification to further disallow visits in nursing homes. Nevertheless, this result depends on the efficacy of vaccines next to our other modelling assumptions. We therefore strongly advise to retain testing capacities at scale, even if they are not needed at a given point in time. In addition, vaccines are highly effective in reducing severe and symptomatic courses of the disease [15,56,57]. Therefore, in situations in which high numbers of employees and residents are vaccinated, purely symptomatic testing within a TTI strategy might lead to a very low number of tests. A point can be made to keep up voluntary mitigation testing in nursing homes, combined with sequencing of samples from positive tests to facilitate the identification of and reaction to novel variants of concern. In addition, regular testing of (partly) vaccinated populations in nursing homes would help to monitor vaccine effectiveness in this cohort in almost real time. Nursing homes lend themselves for such testing and sequencing activity, since they already have established testing infrastructure and medically trained personnel, and new variants of concern are likely to quickly find their way into nursing homes. As such, our results and recommendations are consistent with a recent report by SAGE on the easing of intervention measures in nursing homes [58].

Our model has several limitations: the co-location networks are based on the architecture of nursing home wards, insights of practitioners, and data about occupancy, shared rooms and shared lunch tables at the time of outbreaks and therefore not based on empirical measurements of contacts. We assume that the contact patterns do not change depending on the testing strategy (e.g. contacts to take swab samples). While these assumptions are based on insights from practitioners, these contact patterns are not based on observational data. We do not model the potential replacement of isolated employees by new employees. Neither do we include the possibility of dying from the disease. Therefore, the number of agents in the simulation stays constant throughout the simulation. As simulation durations are short (not longer than six weeks), these simplifications seem warranted. In addition, as the number of agents is small, finite size effects will occur, limiting the size of larger outbreaks. Furthermore, though most model parameters have been calibrated using individual-level observational data, some simplifying assumptions had to be made: all contacts of a given type (e.g. roommates) are assumed to have the same transmission probability, independent of other environmental factors. The viral load dynamics reported in the literature that are translated into a time-dependent transmission risk in the model are approximated in a piece-wise linear way. One could think of test strategies in which the time resolution of our model would need to be increased from days to hours to more accurately assess their effectiveness. We also do not differentiate between agents that have received a single or several vaccination doses and we do not consider the time-dependence of vaccination efficacy. Furthermore, adherence rates to voluntary testing schemes can depend on the test technology used [59,60]. We do not model different adherence rates to voluntary testing and assume a 100% adherence rate in all scenarios. Finally, there are first reports from Austrian nursing homes (J.Z., 2021, unpublished results) that indicate a potentially lower immune response in older people. If these results are substantiated, our already conservative assumptions regarding vaccine efficacy in nursing home residents might have to be reconsidered. It might be interesting to consider different degrees of vulnerability in the residents (e.g. because of comorbidities) to develop ‘personalized’ mitigation testing strategies for specific subgroups, in particular as these residents would be likely to require a level of emergency care that might not be available in a care facility.

In summary, our results indicate that personnel testing twice a week with PCR tests can severely reduce outbreak sizes even without testing of residents and vaccines, provided that other precautionary measures are taken for social interactions of the residents. Antigen tests provide less protection than PCR tests due to their higher detection threshold. On the other hand, vaccines that are moderately effective in preventing infection and transmission render other prevention measures obsolete if at least 80% of residents are vaccinated. Nevertheless, retainment of testing infrastructure, voluntary testing and regular sequencing of positive cases is still beneficial and is advised, in case novel virus variants emerge.

## Endnote

1 Tables are also available in the corresponding data repository at https://doi.org/10.17605/OSF.IO/HYD4R.

## Data accessibility

All data used to calibrate the simulations as well as simulation results and the results of this work in tabular format and all figures are available in the data repository corresponding to this publication at https://doi.org/10.17605/OSF.IO/HYD4R. The simulation package used to model the infection dynamics is openly available [61]. All code used to calibrate the simulations, generate simulation results and generate all figures in this work is available at https://github.com/JanaLasser/nursing_home_SEIRX, the exact code version used for this manuscript has been frozen and is available at https://doi.org/10.5281/zenodo.4898849. Together with the data provided in the data repository at https://doi.org/10.17605/OSF.IO/HYD4R, this allows full reproduction of the results presented in this work.
